# Best practices and opportunities for integrating nutrition specific into nutrition sensitive interventions in fragile contexts: a systematic review

**DOI:** 10.1186/s40795-021-00443-1

**Published:** 2021-07-29

**Authors:** Leila H. Abdullahi, Gilbert K. Rithaa, Bonface Muthomi, Florence Kyallo, Clementina Ngina, Mohamed A. Hassan, Mohamed A. Farah

**Affiliations:** 1 African Institute for Development Policy (AFIDEP), Nairobi, Kenya; 2 Horn Population Research & Development (HPRD), Mogadishu, Somalia; 3 Jomo Kenyatta University of Agriculture and Technology, Nairobi, Kenya; 4 Independent nutrition consultant, Nairobi, Kenya; 5 Independent nutrition consultant, Mogadishu, Somalia; 6 Scaling Up Nutrition (SUN), Office of Prime Minister, Mogadishu, Somalia

**Keywords:** Integration, Nutrition outcome, Nutrition specific, Nutrition sensitive, Multi-sectoral programme

## Abstract

**Background:**

Annually, undernutrition contributes globally to 45% (3.1 million) of preventable deaths in children under 5. Effect following undernutrition i.e. physical growth & cognitive development etc. can be prevented during the first 1000 days also called window of opportunity. There is substantial evidence of positive nutrition outcomes resulting from integrating nutrition-specific interventions into nutrition specific program. However, there is paucity of knowledge on establishing and sustaining effective integration of nutrition intervention in fragile context. The objective of this review is to map and review the integration of nutrition-specific intervention to nutrition sensitive program and its impacts on nutrition outcomes.

**Methods:**

In the study, we systematically searched the literature on integrated nutrition intervention into multi-sectoral programme in PUBMED, Google’s Scholar, the Cochrane Library, World Health Organisation (WHO), United Nations Children’s Fund (UNICEF), World Bank and trial registers from their inception until Oct 30, 2020 for up-to-date published and grey resources. We screened records, extracted data, and assessed risk of bias in duplicates. This study is registered with PROSPERO (CRD42020209730).

**Result:**

Forty-four studies were included in this review, outlining the integration of nutrition-specific interventions among children 0–59 months with various existing programme. Most common integration platform in the study included integrated community case management and Integrated Management of Childhood Illness, Child Health Days, immunization, early child development, and cash transfers. Limited quantitative data were suggestive of some positive impact on nutrition and non-nutrition outcomes with a number of model of integration which varies according to the context and demands of the particular setting in which integration occurs.

**Conclusion:**

Overall, existing evidence for nutrition sensitive and specific interventions is not robust and remains limited. It’s worthwhile to note, for future studies/interventions should be based on the context key criteria like relevance, political support, effectiveness, feasibility, expected contribution to health system strengthening, local capacities, ease of integration and targeting for sustainability, cost effectiveness and financial availability.

**Supplementary Information:**

The online version contains supplementary material available at 10.1186/s40795-021-00443-1.

## Background

Underweight, stunting and wasting are among internationally recognized key indicators that are used to measure nutritional imbalance resulting in undernutrition. Undernutrition is a major cause of disease and death in impoverished communities i.e. fragile settings where sub-optimal growth is responsible for an estimated 2.2 million deaths annually in children under five years of age [1]. In 2018, stunting and wasting affected 149 million and 49 million children, respectively, increasing their susceptibility to mortality from infectious disease [2]. Stunting during childhood can have irreversible, long-term effects, such as decreased adult productivity, depressed cognitive function, and increased risk for obesity and low-birth-weight offspring [3].

Under-nutrition has often been viewed as a problem of limited food availability and solutions for addressing under-nutrition with main focus to increase food production. However, such a vertical approach ignores a wide range of contributing factors which nutrition interventions need to address in order to achieve tangible results. According to the World Health Organization (WHO), integrated health services, also called the ‘horizontal’ approach, represent “the process of bringing together common functions within and between organizations to solve common problems, developing a commitment to shared vision and goals and using common technologies and resources to achieve these goals” [4]. For example, access to safe drinking-water, sanitation and hygiene (WASH) services is a fundamental element of healthy communities and has an important positive impact on nutrition [4]. To have a meaningful WASH & Nutrition integration requires a good understanding of complex causes and determinants of undernutrition.

For the purposes of this document, integration of multi-sectoral approach i.e. food security and livelihood, education, WASH etc. into nutrition intervention is defined broadly as including one or more nutrition specific interventions within a nutrition sensitive intervention or programmatic effort. In this context: nutrition-sensitive interventions are interventions addressing the underlying determinants of fetal and child nutrition and development. The programmes serve as delivery platforms for nutritions pecific interventions, potentially increasing their scale, coverage and effectiveness. For example; food security, adequate care through giving resources at the individual, household and community levels,. Nutrition-specific interventions are interventions addressing the immediate determinants of fetal and child nutrition and development: adequate food and nutrient intake, feeding, care giving and parenting practices, access to clean sanitation environment etc. [5, 6].

**Fig. 1:**
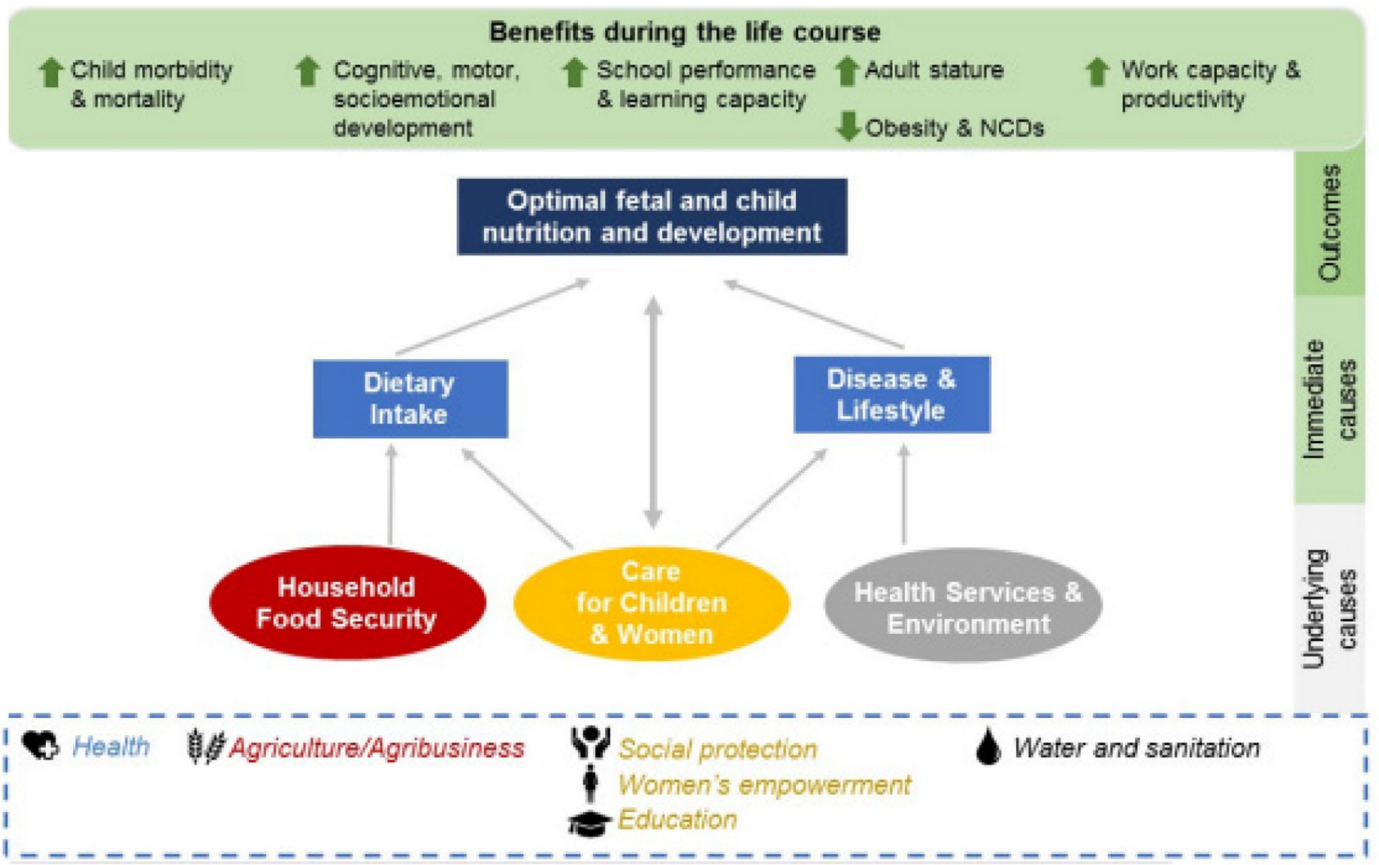
Shows a framework for determinants of nutrition outline the key drivers of malnutrition in society. Adapted from studies entitled ‘Synthesis of Evidence of Multisectoral Approaches for Improved Nutrition’ [7]

Long term and sustainable impact on under-nutrition calls for adopting an integrated multi-sectoral approach. Multi-sectoral program and nutrition integration promotes multi-level response strategies, for example it links curative, preventive and longer term structural actions and acting jointly on existing immediate and underlying causes of under-nutrition as elaborated in the nutrition framework in Fig. 1 below. Some of the nutrition outcome include outcomes related to stunting, wasting, anemia, breastfeeding and low birthweight [7].

Globally, policy makers and implementers need to put in rigorous effort to explore innovative means to reduce the existing high burden of malnutrition [5]. One of the strategies is to strengthen integration of nutrition interventions into existing programmes. Currently there have been significant interest with minimal evidence in integration of nutrition sensitive interventions like agriculture, social safety nets, early child development, classroom education and WASH [6, 8]. Our study proposes to map and synthesis evidence on existing integration platforms with a nutrition lens with an intention to enhance specific nutrition outcomes.

## Broad objective

To synthesize evidence on integration of nutrition-specific and -sensitive interventions in the global context and its applicability in fragile context.

Specific objectives

- Map the existing sector and multi-sectoral nutrition integration platforms.
- Synthesize evidence on best practices for sector and multi-sectoral nutrition integration platforms/programs (both nutrition-specific and sensitive interventions).
- Review evidence on impact of integrated programs on specific nutrition outcomes (such as maternal and child nutrition).
- Identify internal and external drivers of program integrations in different contexts.
- Identify bottle necks to successful sector and multi-sectoral nutrition intervention integration.
- Document opportunities and suggestions to effective program integration of nutrition interventions for fragile context.

## Methods

This study followed the Preferred Reporting Items for Systematic Review and Meta-Analysis Protocols (PRISMA-P) 2015 checklist as indicated in supplementary Table 1 (S1).

S1 Table. PRISMA guideline. S1 Table shows the Preferred Reporting Items for Systematic Reviews and Meta-Analyses.

### Inclusion criteria

#### Types of studies

We included quantitative & qualitative studies describing efforts & approaches to an intervention (integration of services) of a nature including randomized controlled trials (RCTs) and controlled clinical trials (CCTs), or quasi-experimental, controlled before and after studies (CBAs), case studies, policy reports and guidelines.

#### Types of participants

We considered studies/ programme that reported on integration of nutrition sensitive and specific interventions directed at populations with an intention to improve nutrition outcome. The unit of analysis for this review are the programme rather than the individual receiving the intervention. A programme integration is be defined as program that incorporate nutrition specific and sensitive interventions with specific nutrition goals and actions and explicit indicators.

### Study setting

Global settings with applicability to fragile context. The Classification of Fragile and Conflict-Affected Situations as defined by World bank includes:

- Countries with high levels of institutional and social fragility, identified based on publicly available indicators that measure the quality of policy and institutions and manifestations of fragility.
- Countries affected by violent conflict, identified based on a threshold number of conflict-related deaths relative to the population [9].

### Interventions

Integrated management approach, with a focus on holistic and comprehensive nutrition-specific and -sensitive interventions compared to a control. Nutrition specific and sensitive services of particular interest include but not limited to;

- **Nutrition-specific interventions and programmes**
  Promotion of exclusive breastfeeding in the first 6 months
  Promotion of appropriate, adequate and safe complementary feeding for children aged 6–23 months
  Vitamin A supplementation for children aged 6–59 months
  Zinc supplementation for diarrhea management
  Deworming for children from 12 to 59 months
  Iron-folic acid supplementation for pregnant women
  Food fortification of staple foods
  Salt iodization
  Multiple Micronutrient Supplementation (MNPs) for under5s
  Prevention and treatment of moderate acute under-nutrition
  Prevention and treatment of severe acute malnutrition
  Dietary diversity among pregnant and lactating mothers
  Adolescent health and preconception nutrition

Nutrition-specific interventions aim to address the more immediate causes of undernutrition, such as inadequate dietary intake and poor health.

b) **Nutrition-sensitive interventions**
  Agriculture and food security
  Social protection (social safety nets programs such as CVAs, Food Donations/Aids, NHIF, CT)
  Early childhood development and education (ECDE) (This will include child stimulation play and responsiveness, Nutrition)
  Maternal mental health
  Women’s empowerment
  Child protection
  Water and sanitation (WASH)
  Health and family planning services
  Schooling

Nutrition-sensitive interventions address the underlying and basic causes of undernutrition (e.g. poverty, food insecurity, education, women’s empowerment, and social status) through indirect but plausible pathways. Nutrition-sensitive interventions can also serve as delivery platforms for nutrition-specific interventions [6, 10].

### Comparison group

Program or group with non-integrated nutrition services. **Types of integration outcome**

1. Integrated programme characteristics to include:

- The programme start year, location(s) & duration;
- Level of programme integration at which implemented I.e. primary care, secondary care, tertiary care, and quaternary care (teaching and referral hospitals), public / private sector;
- Whether the integration covers specific groups e.g. adults’ vs children, pregnant and lactating women, under-fives, adolescents etc...
- Types of services /intervention integrated.
- What were the components of the integration process? i.e. was it joint programme where clients were seen for example on the same day, or was it just referral pathways between the services.
  2. Programme integration: We will assess how the approach to integration was developed and designed i.e.
- How the integration of nutrition sensitive and specific interventions was executed;
- Challenges and barriers linked to the programme integration;
- Facilitators of programme integration.
  3. Programme results
**-**What is the impact of integration broadly categorized as;

- Impact on target group nutrition outcome
- Impact on other key client-centred outcomes E.g. Number of client visits required, client satisfaction
- Impact on nutrition and health of households

### Type of nutrition outcomes

Stunting, wasting, anemia, breastfeeding and low birthweight

#### Exclusion criteria

We excluded studies evaluating the impact of stand-alone programmes on nutrition outcomes.

### Search methods for identification of studies

We developed a comprehensive search strategy from their inception until Oct 30, 2020 using the framework described in Supplementary Table 2 (S2), for websites, peer-reviewed studies and grey literature with no time and language limits. The following databases was included at a minimum: PUBMED, Google’s Scholar database and the Cochrane Library. We searched the websites of the World Health Organisation (WHO), United Nations Children’s Fund (UNICEF), World Bank and trial registers such as the International Clinical Trials Registry Platform (ICTRP) for trials. Furthermore, we screened the reference lists of all the included studies and related systematic reviews for other potentially eligible primary studies.

S2 Table. Search term in PubMed. S2 Table shows the detailed search term used in PubMed in the study.

### Data collection and analysis

Two authors independently screened through titles and abstracts of the retrieved records to identify potentially eligible studies. The full texts of the potentially eligible studies was assessed using the pre-specified eligibility criteria. The two authors compared lists of included studies and resolved discrepancies by discussion and consensus. Disagreements was resolved through discussion and a third author was contacted when the authors failed to reach consensus.

### Data extraction and management

A data collection form was designed and used independently by two review authors to extract data from the included studies. The following information was extracted from each included study; study setting (region/site and country), type of study, study participants, types and description of the intervention and study outcomes, as described above.

### Risk of bias (quality) assessment

The Cochrane Collaboration’s risk of bias tool was used for cluster and individual randomized controlled trials [11] and for non-randomized studies, the risk of bias in non-randomized studies of interventions (ROBINS-I) tool was used [12]. On the other hand, the quantitative observational risk of bias for cohort and cross-sectional studies and qualitative risk of bias for qualitative studies was assessed using CASP tool as relevant [13].

### Subgroup analysis

The following considerations was taken during subgroup analysis of review data: study design, level / sector of the programme at which integration performed, types of services integrated i.e. nutrition specific and sensitive service delivery, the intervention approaches/strategies used.

### Assessment of heterogeneity

For quantitative studies of similar interventions reporting similar outcomes, statistical heterogeneity was examined using the chi-squared test for homogeneity (with significance defined at 10% alpha level). Statistical heterogeneity was quantified using the I^2^statistic. For qualitative studies or qualitative outcomes, heterogeneity was discussed in the text only.

### Data synthesis

We described data using standard summary statistics and perform meta-analysis when more than 3 studies for each outcome meet the criteria for the systematic review. Where the outcomes of interest were either dichotomous or continuous; we calculated risk ratios and their corresponding 95% confidence intervals and *p*-values for dichotomous outcomes, and mean differences and standard deviations for continuous outcomes. Where outcomes are measured using different scales, we calculated standardised mean differences (SMD). A random effects model was used with the assumption that the true effect size varied between studies. For the outcome measure that were qualitative in format i.e. patient satisfaction that cannot be quantified, we discussed it narratively.

### Quality assessments

Due to the nature of the study where we assessed the level of integration at the programme level we were not able to assess the overall quality of evidence hence, we did not assess the quality of evidence in this review.

## Results

### Results of the search

**Fig. 2:**
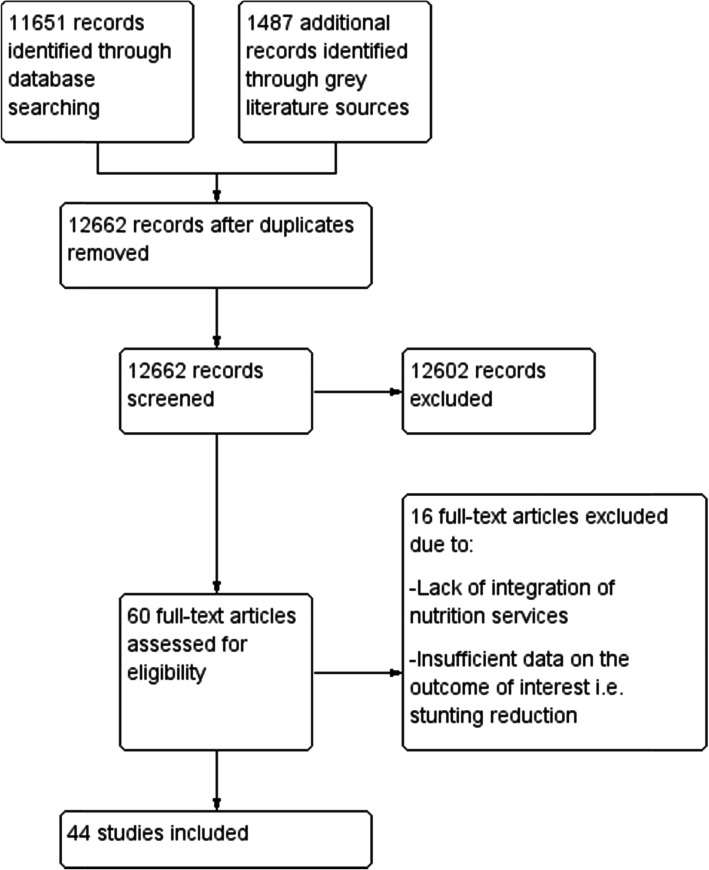
Prisma flow diagram. shows the process of selecting relevant studies from 13,138 records. After removing 476 duplicates, 12,662 records were screened; 12,602 of the records were excluded based on the title and abstract. Full texts of 60 potential eligible articles were retrieved and reviewed for inclusion. Of the 60 records, 44 studies met our inclusion criteria and 16 studies were excluded

We identified 13,138 records from the electronic databases and grey sources. After excluding 476 duplicates, we screened 12,662 records, and found that 12,602 records were not relevant to our review question. We reviewed the remaining 60 potentially eligible full-text articles for inclusion and excluded 16 of them with reasons listed in Fig. 2. Forty-four studies met the inclusion criteria and were described in Table 1 below. The search process and selection of studies is presented in the Prisma flow diagram Fig. 2 below.

**Table 1:** Characteristics of included studies

Study ID	Country	Study design	Duration of intervention	Integration program	Nutrition Interventions Included
Arifeen et al. 2009 [14]	Bangladesh	Cluster RCT	2 years	Nutrition into IMCI/iCCM	Counselling of mothers on breastfeeding and appropriate complementary feeding, local feeding practices, growth monitoring, supplementary nutrition, vitamin A supplementation, and screening, management and referral for malnutrition.
Armstrong et al. 2004 [15]	Tanzania	Cross-sectional study	Not stated		
Bhandari et al. 2012 [16]	India	Cluster RCT	3 years and 4 months		
Bryce et al. 2005 [17]	Tanzania	Non-RCT	1 year		
El Arifeen et al. 2004 [18]	Bangladesh	Cluster RCT	2 years		
Friedman & Wolfheim 2014 [19]	Multi-countries	Mixed studies	Not stated		
Masanja et al. 2005 [20]	Tanzania	Cross-sectional study	Not stated		
Mazumder et al. 2014 [21]	India	Cluster RCT	3 years and 4 months		
Miller et al. 2014 [22]	Ethiopia	Cross-sectional study	1 year		
Rasanathan et al. 2014 [23]	Sub-Saharan countries	Cross-sectional study	Not stated		
Schellenberg et al. 2004 [24]	Tanzania	Cross-sectional study	Not stated		
Taneja et al. 2015 [25]	India	Cluster RCT	1 year		
Aguayo et al. 2013 [26]	India	Cross-sectional study	1 year	SAM/MAM into Health Services	Community and facility-based management of SAM and MAM.
Amadi et al. 2016 [27]	Zambia	Cohort study	3 years		
Brits et al. 2017 [28]	South Africa	Cohort study	1 year		
Deconinck et al. 2016 [29]	Niger	Qualitative study	Not stated		
Kouam et al. 2014 [30]	Bangladesh	Qualitative study	Not stated		
Puett et al. 2015 [31]	Bangladesh	Qualitative study	Not stated		
Puett et al. 2013 [32]	Bangladesh	Mixed study	Not stated		
Sadler et al. 2011 [33]	Bangladesh	Cross-sectional study	Not stated		
Tadesse et al. 2017 [34]	Ethiopia	Cohort study	14 weeks		
Doherty et al. 2010 [35]	Ethiopia, Madagascar, Tanzania, Uganda, Zambia, Zimbabwe	Cross-sectional study	6 months	Nutrition into Child Health Days	Vitamin A supplementation and nutrition screening.
Palmer et al. 2013 [36]	Multi-countries	Cross-sectional study	Not stated		
Anand et al. 2012 [37]	28 sub-Saharan African countries	Cross-sectional study	Not stated	Nutrition into Immunization	Vitamin A supplementation, early and exclusive breastfeeding, infant and young child feeding practices and growth monitoring.
Baqui et al. 2008 [38]	India	Quasi-experimental	3 years		
Ching et al. 2000 [39]	Philippines and Vietnam	Cross-sectional study	Not stated		
Hodges et al. 2015 [40]	Sierra Leone	Quasi-experimental	6 months		
Klemm et al. 1996 [41]	Philippines	Cross-sectional study	6 months		
Ropero-Álvarez et al. 2012 [42]	Multi-countries	Cross-sectional study	Not stated		
Fernandez-Rao et al. 2014 [43]	India	RCT	1 year	Nutrition into ECD	Home/preschool fortification with multiple micronutrient powder, responsive stimulation, early nutrition interventions, monitoring of child nutrition and growth promotion.
Gowani et al. 2014 [44]	Pakistan	RCT	2 years and 7 months		
Yousafzai et al. 2014 [45]	India	RCT	1 year		
Grellety et al. 2017 [46]	Congo	RCT	6 months	Nutrition into Cash Transfer Programs	Treatment of SAM according to the national protocol and counselling with or without a cash supplement of US$40 monthly for 6 months.
Berti et al. 2010 [47]	Ethiopia, Ghana, Malawi & Tanzania	Cross-sectional survey	10 years	Nutrition into Other Programs	Infant and young child feeding practices and micronutrient supplementation.
Fagerli et al. 2017 [48]	Kenya	Cross-sectional study	1 year		
Grossmann et al. 2015 [49]	Guatamala	Before and after study	3 months		
Guyon et al. 2009 [50]	Madagascar	Before and after study	5 years		
Nguyen et al. 2017 [51]	Bangladesh	Cluster-RCT	2 years		
Parikh et al. 2010 [52]	Dominican Republic	Cross-sectional study	1 year		
Saiyed & Seshadri 2000 [53]	India	Cross-sectional study	Not stated		
Singh et al. 2017 [54]	India	Quasi experimental	18 months		
Sivanesan et al. 2016 [55]	India	Cross-sectional study	Not stated		
Tandon, 1989 [56]	India	Cross-sectional study	Not stated		
Head Jeniffer 1999 [57]	Ethiopia	Cross-sectional study	Not stated		

Table 1 shows a summary of the included studies for integrated programs and nutrition intervention involved for each study i.e. study settings

### Study description and geographical location

We included 44 papers that met the inclusion criteria. Studies ranged from individual randomized control trials, Cluster RCT, cohort, cross-sectional studies, to qualitative studies. The studies were representative from wide range of countries in four continents i.e. Asia (India, Bangladesh, Philippines, Vietnam, Pakistan); Africa (Congo, Sierra Leone, Ethiopia, Zambia, Madagascar, Malawi, Ghana, Niger, South Africa, Uganda, Tanzania, Kenya); North America (Dominican Republic); South America (Guatamala). Most of the quantitative studies reported the duration of the intervention to range from 14 weeks to 10 years. The median time of intervention was 1 year.

### Nutrition integration platform

We reviewed and mapped 44 included studies according to the primary programmes into which nutrition-specific interventions were integrated. These primary programmes, or “integration platforms,” included integrating nutrition into following existing program:

- Integrated Management of Childhood Illness and integrated community case management (IMCI/iCCM),
- Integrating management of severe and moderate acute malnutrition (SAM/MAM) into health services,
- Integrating nutrition into Child Health Days (CHD) and integrating nutrition into immunization,
- Integrating nutrition into social programmes, including Early Childhood Development (ECD) and cash transfers.
- Other programmes;” i.e. programmes that integrated nutrition-specific interventions, including promotion of breastfeeding and appropriate complementary feeding, feeding practices, growth monitoring, supplementary nutrition, vitamin A supplementation, home fortification, screening and management for malnutrition into existing community health facilities.

### Risk of bias

Of the 10 randomized control study, all the studies were having moderate risk of bias due to inadequate sequence generation and allocation concealment, as well as the lack of blinding of the participants and personnel and blinding of the outcome assessor. Blinding could not be achieved due to the nature of the intervention. Amongst 6 non-randomized control studies, the risk of bias was moderate as most of the domains on the risk of bias assessment were elaborated to be with minimal flaws. For the quantitative 25 observational studies most of the studies risk of bias was ranging from moderate to low as most of the domains on the risk of bias assessment were elaborated to be with some flaws. Additionally, 3 studies were qualitative studies with low risk of bias due to flaws on the domain of risk of bias as shown in supplementary Table 3.

### Impact of integration models or approaches on nutrition integration following nutrition interventions

#### Integrated nutrition intervention and IMCI/iCCM programmes

**Fig. 3:**
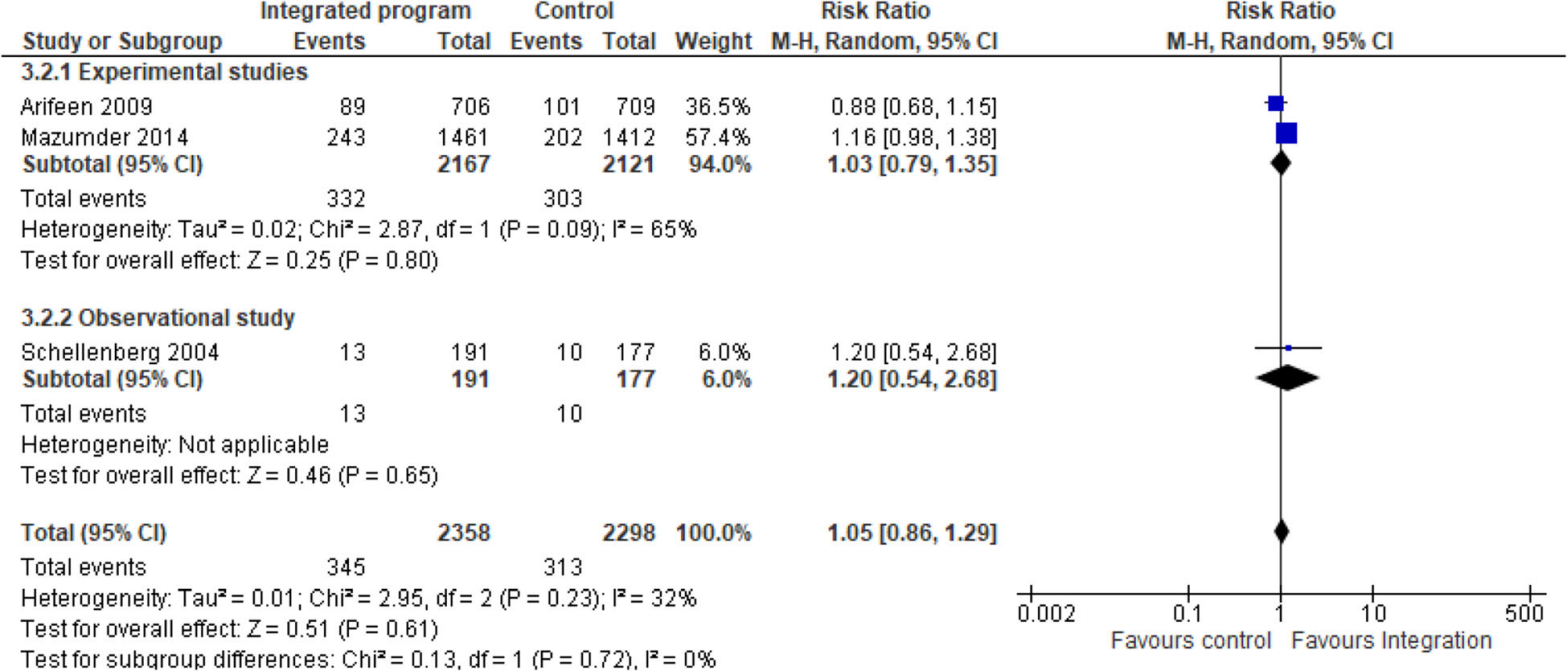
Integrated program on complementary feeding nutrition intervention. Shows the effect of integrated program on complimentary feeding practices compared to the non-integrated program among children aged 6–9 months

**Fig. 4:**
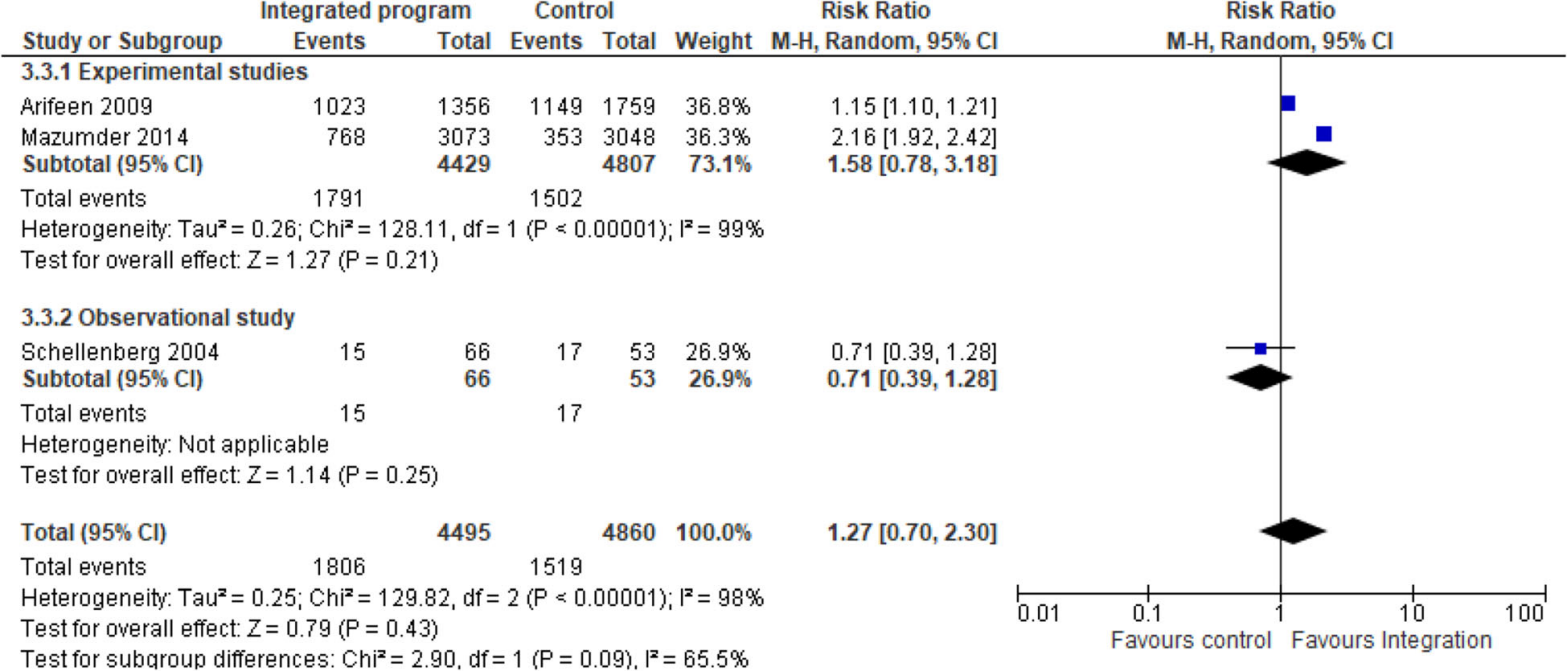
Integrated program on exclusive breastfeeding nutrition intervention. Shows the effect of integrated program on exclusive breastfeeding practices among children younger than 6 months compared to the non-integrated program

**Fig. 5:**
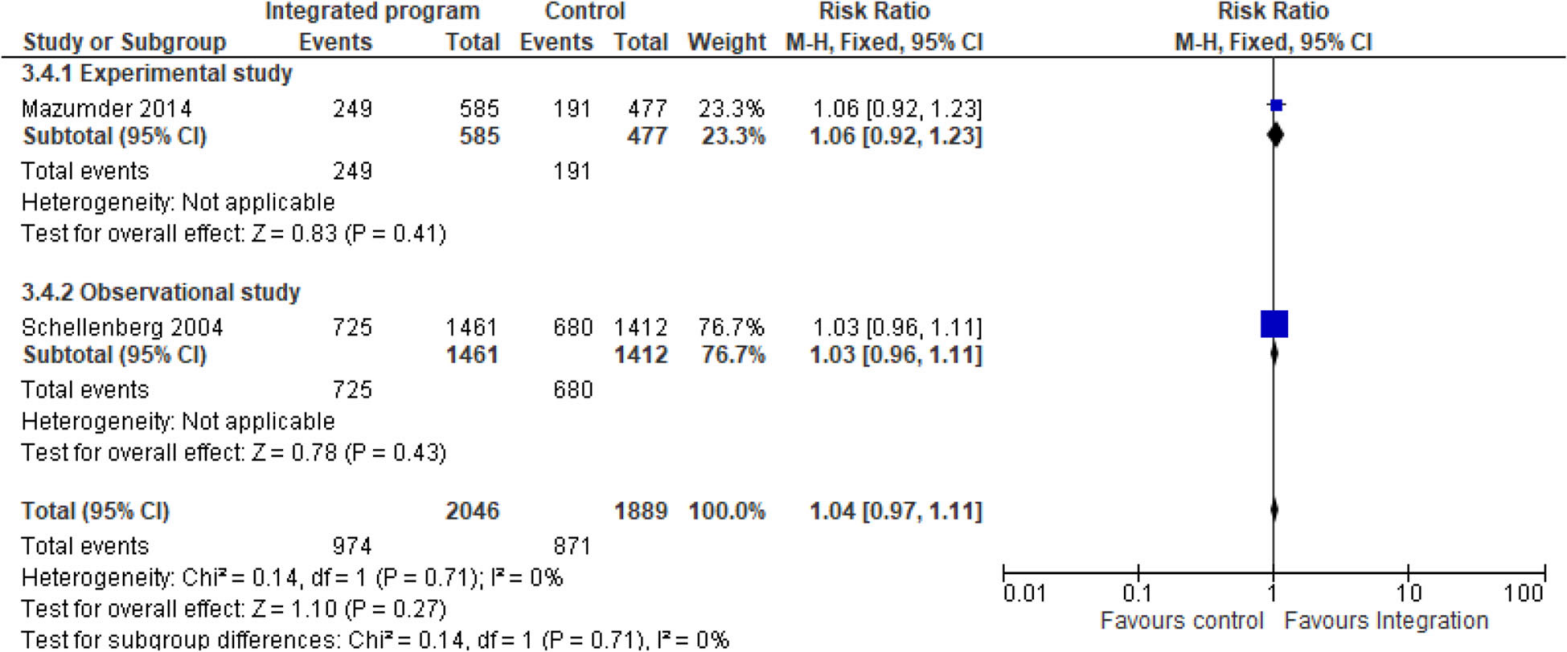
Integrated program on stunting. Shows the effect of integrated program in stunting among children aged 24–59 months compared to the non-integrated program

**Fig. 6:**
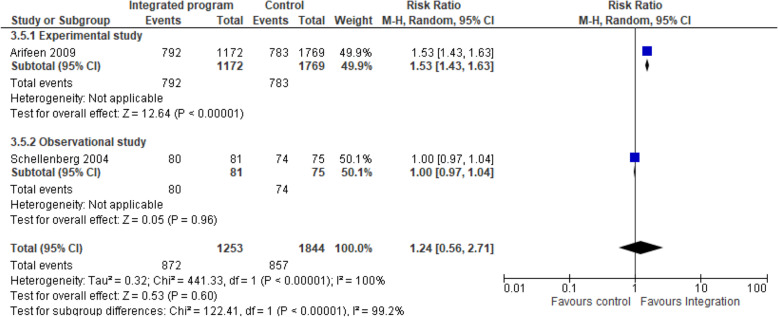
Integrated program on wasting. Shows the effect of integrated program on wasting among children aged 0–23 months (<− 2 WHZ) compared to the non-integrated program

- Integrated nutrition intervention and IMCI/iCCM programmes on complementary feeding: Three studies [14, 21, 24] pooled analysis of nutrition-specific intervention suggests that the effect of integrated program enhanced the complimentary feeding practices by 5% compared to the non-integrated program (RR 1.05, 95% CI 0.86 to 1.29; I^2^ 0%; 5314 participants). A subgroup analysis showed low heterogeneity in the effect of integration on complimentary feeding practices among the group Fig. 3. Complimentary feeding practices targeted children aged 6–9 months receiving breast milk and complementary feeding. The nutrition specific intervention included counselling of mothers on breastfeeding and appropriate complementary feeding, local feeding practices, growth monitoring, supplementary nutrition, vitamin A supplementation, and screening, management and referral for malnutrition.

b) Integrated nutrition intervention and IMCI/iCCM programmes on exclusive breastfeeding: Three studies [14, 21, 24] pooled analysis of nutrition-specific intervention suggests that the effect of integrated program enhanced the exclusive breastfeeding practices among children younger than 6 months by 27% compared to the non-integrated program and the effect showed a statistically significant difference among the integrated group (RR 1.27, 95% CI 0.70 to 2.30; I^2^ 65.5%; 12,680 participants) Fig. 4. A subgroup analysis showed high heterogeneity hence the results should be interpreted with caution. The nutrition specific intervention included counselling of mothers on breastfeeding, local feeding practices, growth monitoring, supplementary nutrition, vitamin A supplementation, and screening, management and referral for malnutrition.

c) Integrated nutrition intervention and IMCI/iCCM programmes on stunting: Two studies [21, 24] pooled analysis of nutrition-specific intervention suggests that integrated program had minimal protective effect in stunting among children aged 24–59 months compared to the non-integrated program (RR 1.04, 95% CI 0.97 to 1.11; I^2^ 0%; 5780 participants) Fig. 5. A subgroup analysis showed low heterogeneity on the effect in stunting. The nutrition specific intervention included counselling of mothers on breastfeeding and appropriate complementary feeding, local feeding practices, growth monitoring, supplementary nutrition, vitamin A supplementation, and screening, management and referral for malnutrition.

d) Integrated nutrition intervention and IMCI/iCCM programmes on wasting: Two studies [21, 24] pooled analysis of nutrition-specific intervention suggests that integrated program had no protective effect in wasting among children aged 0–23 months (<− 2 WHZ) compared to the non-integrated program (RR 1.24, 95% CI 0.56 to 2.71; I^2^ 99.2%; 4826 participants) Fig. 6. A subgroup analysis showed high heterogeneity hence the results should be interpreted with caution. The nutrition specific intervention included counselling of mothers on breastfeeding and appropriate complementary feeding, local feeding practices, growth monitoring, supplementary nutrition, vitamin A supplementation, and screening, management and referral for malnutrition.

#### Integrated nutrition intervention and immunisation programmes

**Fig. 7:**
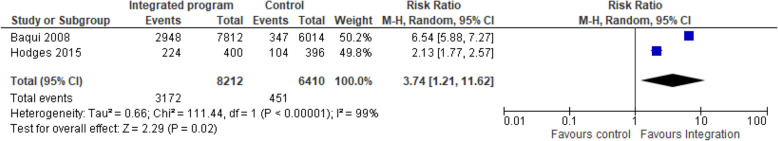
Integrated program on breastfeeding initiation. Shows the effect of integrated program on early breastfeeding initiation practices within 1 h of delivery compared to the non-integrated program

**Fig. 8:**
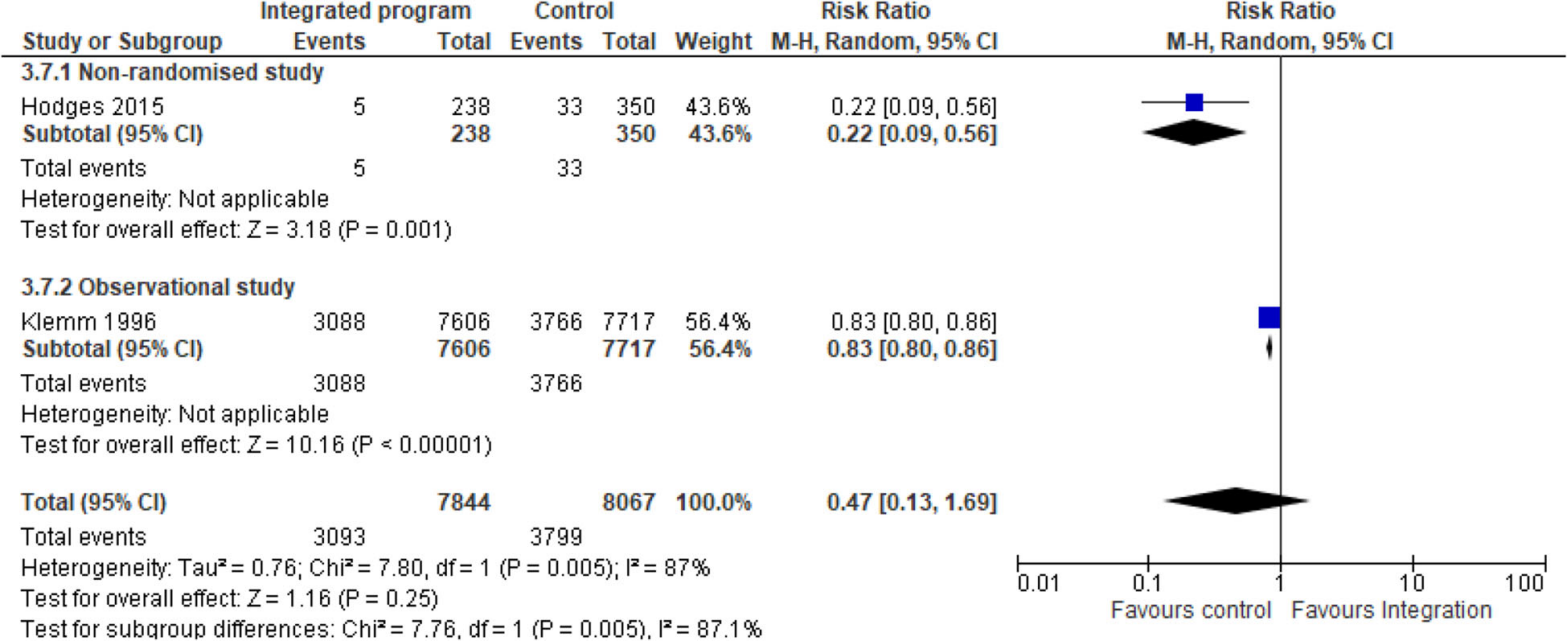
Integrated program on underweight. Shows the effect of integrated program toward underweight of children > 2 years compared to the non-integrated program

e) Integrated nutrition intervention and immunisation programmes on initiated breastfeeding within first hour: Two studies [38, 40] pooled analysis of nutrition-specific intervention suggests that the effect of integrated program enhanced the early breastfeeding initiation practices within 1 h of delivery by 3 folds compared to the non-integrated program (RR 3.74, 95% CI 1.21 to 11.62; I^2^ 99%; 18,245 participants) Fig. 7. A subgroup analysis showed high heterogeneity hence the results should be interpreted with caution. The nutrition specific intervention included Vitamin A supplementation, early and exclusive breastfeeding, infant and young child feeding practices and growth monitoring.

f) Integrated nutrition intervention and immunisation programmes on underweight: Two studies [40, 41] pooled analysis of nutrition-specific intervention suggests that the effect of integrated program was protective toward underweight of children > 2 years by 53% compared to the non-integrated program and the effect showed a statistically significant difference among the integrated group (RR 0.47, 95% CI 0.13 to 1.69; I^2^ 87.1%; 22,803 participants) Fig. 8. A subgroup analysis showed high heterogeneity hence the results should be interpreted with caution. The nutrition specific intervention included Vitamin A supplementation, early and exclusive breastfeeding, infant and young child feeding practices and growth monitoring.

Two platforms (CHD and ECD programmes) did not have sufficient data for quantitative analysis of outcomes. Table 1 summarizes the estimates for the pooled outcomes reported as we could not conduct a meta-analysis for any of the nutrition-specific or non-nutrition outcomes where studies were one-time cross-sectional surveys and did not provide data for comparison.

For integrated SAM/MAM programmes, recovery from SAM was reported to range from 18% in a facility-based management programme in India to 23% in the primary care health care system in Ethiopia, 50% in South Africa, 65% in the community component in India, and 70% in Zambia [26–28, 34]. In the integrated Zambia programme, recovery from MAM was demonstrated to be around 80%, and the study reported an impact on SAM case fatality rates [27]. A single study on integrated nutrition and cash transfer programmes [46] reported higher SAM recovery and a lower MAM, and lower SAM relapse in the integrated group compared with the control group. Change in weight, weight for age z score, weight for height z score, and body mass index z score were also better in the intervention group compared with the control group. The study reported to have no difference in change in height/length, height/age, or mid-upper arm circumference between intervention and control groups.

There were other integrated nutrition and other programmes that could not be categorized in the above platforms and integrated nutrition-specific interventions. The programmes includes promotion of breastfeeding and appropriate complementary feeding, feeding practices, growth monitoring, supplementary nutrition, vitamin A supplementation, home fortification, screening and management for malnutrition into existing community health setups, and maternal, newborn, and child health centres and clinics). The studies were one time cross-sectional surveys hence we could not pool any of the outcomes.

Narratively, among nutrition-specific outcomes, the India programme showed improved early initiation of breastfeeding and exclusive breastfeeding [54], and programmes for Kenya and Bangladesh suggested higher intervention coverage for vitamin A supplementation, paediatric iron folic acid supplementation, and supplementary nutrition [48, 51]. The Kenya programme also reported significant increase in the exclusive breastfeeding rates from baseline to end line, as well as improved antenatal visits, health facility delivery, and postnatal visits [48].

#### Best practices, drivers and bottlenecks to integration with applicability to fragile context

A growing body of evidence supports the notion that integration of nutrition sensitive programs and nutrition specific interventions provide stronger impacts on nutritional and non-nutritional outcomes than either intervention alone. Combined interventions may be more efficient than separate interventions, because they are intended for the same population and make use of the same facilities, transportation, and client contacts. In addition, for families, particularly for those most at risk, combined interventions can also lead to increased access to services. In the included studies table two below summarises the findings and opportunities or barriers that were observed in eight studies during integration of nutrition interventions to various program. Thematically some of the key drivers/ opportunities that facilitated, and barriers that hindered, integration can be summarized as Table 2 below.

**Table 2:** bottle neck and opportunities associated with best practices on integration model with applicability to fragile context

Study ID/Country	Integration program/ Intervention	Key findings/ Recommendations	Barriers and opportunities for improvement
Armstrong et al. 2004 [15] Tanzania	Nutrition into IMCI/ICCM **Intervention**: Counselling of mothers on breastfeeding and appropriate complementary feeding, local feeding practices, growth monitoring, supplementary nutrition, vitamin A supplementation, and screening, management and referral for malnutrition	There were few differences between IMCI and comparison districts in the level of health system support for child health services at facility level.	**Opportunities**: IMCI, in the presence of a decentralized health system with practical health system planning tools, is feasible for implementation in resource poor countries and can lead to rapid gains in the quality of case-management.
Bhandari et al. 2012 [16] India		Implementation of the IMNCI resulted in substantial improvement in infant survival and in neonatal survival in those born at home.	**Opportunities**: High quality training, ensuring adequate supervision, timely supplies, and task based incentives to community health workers was critical for the observed effect.
Aguayo et al. 2013 [26] India	SAM/MAM into Health Services **Intervention**: Community and facility-based management of SAM and MAM.	The survival rates in the integrated model for the management of SAM (IM-SAM) program were very high	**Opportunities**: Existing health systems can be strengthened with feasible adjustments i.e. integrated model that comprises facility- and community-based therapeutic care
Amadi et al. 2016 [27] Zambia		Comprehensive community malnutrition programme, incorporating HIV care, can achieve low mortality	**Opportunities**: Community-based screening may seem like a resource-intensive approach but the result is justified
Brits et al. 2017 [28] South Africa		Half of the children improved from severe malnutrition to underweight or exited at target weight	**Barriers** observed include; obstacles in implementing the guidelines correctly and lack of monitoring of the integrated program.
Deconinck et al. 2016 [29] Niger		Key hindering factors identified were not fully understanding severity, causes and consequences of the problem	**Barriers**: lack of information on burden of acute malnutrition, recognition of the public health priority, leadership for policy adaptations and implementation, technical and financial resources, effectiveness of the intervention and capabilities and motivation of health actors.
Baqui et al. 2008 [38] India	Nutrition into Immunization **Intervention**: Vitamin A supplementation, early and exclusive breastfeeding, infant and young child feeding practices and growth monitoring.	Most of the reduction in mortality was in the group who were visited within the first 3 days of birth	**Opportunitie**s: Reaching newborn babies at the community level is crucial in settings where the availability and utilization of facility-based care is low. Systems must also be put in place to ensure that these workers visit neonates at home during the first hours and days after birth and provide a link to competent health services **Barrier**s: Workers’ competency in the new neonatal component of the programme, their workload and inadequate management and supervision were possible barriers to higher coverage.
Fagerli et al. 2017 [48] Kenya	Nutrition into Other Programs **Intervention**: Infant and young child feeding practices and micronutrient supplementation.	The study shows multi-sectoral integration including hygiene, nutritional, clean delivery incentives, higher education level, and geographical contiguity to health facility were associated with increased use of maternal health services by pregnant women.	**Barriers**: low education level, distance from health facilities, and poor socioeconomic status.

Table 2 shows a summary of the included studies with their key recommendation and potential barriers and opportunities to integration

Key drivers/opportunities that facilitated the integration were:

- Broad context: political readiness, interest, and support and progress monitoring for resilience and development initiatives
- Nature of the problem: knowledge of causes and consequences of illness and prevention and treatment pathways, accurate information on the burden of disease, and political and social environment to recognize the problem and initiate change
- Intervention: skill development; decentralised care to increase staff exposure to the breadth of the health care system, access, utilization and involvement; quality of care showing effectiveness and increasing awareness and user satisfaction; and clinical, organizational and management capacities in successful sites
- Adoption system: compatibility with personal, professional and institutional goals, values and principles; collaborative support, engagement and involvement; learning and career development opportunities; and support for problem solving
- Health system characteristics: policy adaptation and translation; expanded, regulated and aligned partnerships; expanded health workforce; and decentralised care

Key barriers that hindered the integration were:

- Broad context: demographic pressure and multi-sectoral approach diverting a sectoral focus
- There is lack of evidence on the nature of the problem
- Intervention: clinical, organizational and management capacity gaps in certain sites, interventions substituted by partners and limited community awareness and involvement reinforcing mistrust
- Adoption system: partner support favouring evading responsibility; lack of interest or motivation or collaboration in care and learning, feeling of curtailed career development, and high workload
- Health system characteristics: multiple health information systems; underfunded health budget; short-term emergency funding; high staff turnover and attrition; limited logistic capacity for bulky, expensive supplies; and limited community and patient/ care giver involvement and empowerment

## Discussion

The comprehensive review included 44 articles from the identified 13,138 records. From the study, most of the quantitative studies ranging from RCT to cohort have assessed the intervention over different range of time. Majority of the study have assessed the study over the period of 1 year with some study having least period of 14 weeks and longest period of 10 years. Where applicable, we conducted subgroup analysis by study design and we observed that the evidence from observational studies is going in the same direction as experimental studies. Hence no much difference on the findings based on study design. Majority of the study design conducted in this humanitarian context were cross-sectional studies with low quality of evidence, the study findings need to be interpreted with caution.

Nutrition-specific interventions as defined in the introduction aims to address the more immediate causes of undernutrition, such as inadequate dietary intake and poor health [6, 10]. Evidence suggest that nutrition-specific intervention could have a dramatic impact on reducing malnutrition. However, nutrition-specific interventions alone will not eliminate undernutrition; rather, in combination with nutrition-sensitive interventions, there is enormous potential to enhance the effectiveness of nutrition investments worldwide.

On the other hand, nutrition-sensitive interventions address the underlying and basic causes of undernutrition (e.g. poverty, food insecurity, education, women’s empowerment, and social status) through indirect but plausible pathways [6, 10]. Interventions such as agriculture, livelihoods, social safety nets, women’s empowerment, education, and early child development, all contribute indirectly to improving nutrition outcomes. Nutrition-sensitive interventions can also serve as delivery platforms for nutrition-specific interventions. Harmonisation of interventions and messages across community platforms of different sectors is crucial for coherence.

Combined interventions may be more efficient than separate interventions, because they are intended for the same population and make use of the same facilities, transportation, and client contacts. However, in order for integrated nutrition to be embedded to multi-sectoral program successfully, a variety of opportunities and challenges must be addressed. From an intervention perspective, the key to successful integration was evidence-based strategy; from a program perspective, it was leadership, capacities and resources; from an adoption system perspective, it was knowledge, capabilities, motivation and opportunities to provide quality interventions; and from the broader context perspective, it was political interest and recognized need. Key challenges that need to be addressed include workload of staff and supervisors, communication and coordination among different integrated programmes and among staff in different sectors, and an acknowledgement at the national and community levels that comprehensive address both nutrition and non-nutrition outcome.

Our systematic review shows that evidence on the benefits of integration of fragile context on nutrition interventions is limited and too weak to allow for clear conclusions about when either approach is desirable. The limited evidence available suggests that integrated approaches compared with unintegrated approaches, improve outcome however, this should be interpreted with caution. From the study, it is evident that heterogeneity could be due to many reasons i.e. intervention duration, study design and subjects. So far, we were able to account for the heterogeneity due to study design which was reported. However, this should be taken into consideration when interpreting evidence.

In addition, following the obtained evidence, there is currently a great interest and need to document the true costs and benefits of integrating interventions for young children across relevant sectors and building on existing community resources. However, at present, there is paucity of data on this important element of integrated programming and most importantly in fragile context. Hence a need for a robust evidence to address the need.

Most importantly, the prioritisation of interventions in any context should be based on a robust situational analysis supported by strong evidence. Despite strong associations and plausible impact pathways between nutrition intervention and outcomes, the existing evidence base for some nutrition interventions, especially nutrition sensitive approaches, remains limited. Evidence suggest that prioritisation of integrated nutrition interventions in fragile context is strongly dependent on the following criteria: relevance, political support, effectiveness, feasibility, expected contribution to health system strengthening, local capacities, ease of integration and targeting for sustainability, cost effectiveness, and dependent on available financing and presence of a funding gap.

Investments in the generation of robust and relevant evidence to inform implementation of nutrition interventions are crucial to ensure optimal nutrition impact, strengthen accountability and guide the evolution of policies. Ensuring the incorporation of both high impact nutrition specific interventions and essential nutrition sensitive intervention areas in the multi-sector need to be understood as a key component of any broader national commitment and multi-sectoral strategic framework for eradicating malnutrition through a rights-based approach.

## Conclusion

Combined interventions may be more efficient in integration of nutrition intervention into multi-sectoral program. For example, a comprehensive package not limited to; hygiene, nutritional services, clean delivery incentives, awareness and education, and distance to services motivated an increase in the use of services. Over and above, community-level nutrition integration actions show the breadth and variety of nutrition-related positive outcomes across the studies.

## Recommendations

There is scarce data around integrated nutrition programmes in fragile context. Either way in non-fragile context evidence reveal mixed evidence and information gaps. The evidence does suggest, however, that there is much potential for integrating nutrition interventions into related programmes to ensure adequate, efficient service delivery, and impact on nutrition outcome. We recommend that context-specific learning of integrating malnutrition may expand to include causal modelling and scenario testing to inform strategy designs. The method may also be applied to monitor progress of integrating nutrition by the multi-sectoral nutrition plan to guide change.

## Supplementary Information

**Additional file 1: Table S1.** PRISMA guideline. **Table S2.** Pubmed search. **Table S3.** Risk of bias of the included studies.

## Data Availability

This is a systematic review and all the included studies/data are elaborated in the findings.

## Abbreviations

CBAs: Controlled before and after studies
CCTs: Controlled clinical trials
CHD: Child Health Days
ECD: Early Childhood Development
ECDE: Early childhood development and education
FSI: Fragile States Index
GRADE: Grading of Recommendations, Assessment, Development and Evaluations
ICCM: Integrated Community Case Manage
IMCI: Integrated Management of Childhood Illnesses
ICTRP: International Clinical Trials Registry Platform
IYCF: Infant and Young Child Feeding
MAM: Moderate Acute Malnutrition
MNPs: Multiple Micronutrient Supplementation
PRISMA-P: Preferred Reporting Items for Systematic Review and Meta-Analysis Protocols
RCTs: Randomized controlled trials
ROBINS-I: Risk of bias in non-randomized studies of interventions
SAM: Severe Acute Malnutrition
SMD: Standardised mean differences
SUN-FP: Scaling Up Nutrition -Focal Person
UNICEF: United Nations Children Fund
WASH: Water Hygiene and Sanitation
WHO: World Health Organization

## References

[1] Black RE, Allen LH, Bhutta ZA, Caulfield LE, de Onis M, Ezzati M (2008). Mathers C and J Rivera maternal and child undernutrition: global and regional exposures and health consequences. Lancet.

[2] World Health Organization (2019). WHO Global Database on Child Growth and Malnutrition.

[3] Victora CG, Adair L, Fall C, Hallal PC, Martorell R, Richter L, Sachdev HS (2008). Maternal and child undernutrition: consequences for adult health and human capital. Lancet.

[4] World Health Organization Study Group on Integration of Health Care Delivery (1996). Integration of health care delivery.

[5] Horton R, Lo S (2013). Nutrition: A quintessential sustainable development goal. Lancet..

[6] Ruel M (2013). Nutrition-sensitive interventions and Programmes: how can they help to accelerate Progress in improving maternal and child nutrition. Lancet.

[7] Horton S, Mbuya M, Wilkinson C (2017). Synthesis of Evidence of Multisectoral Approaches for Improved Nutrition.

[8] Gillespie S, Haddad L, Mannar V, Menon P, Nisbett N (2013). Maternal and child nutrition study group. The politics of reducing malnutrition: building commitment and accelerating progress. Lancet..

[9] The World Bank (2020). The Fund for Peace. Fragile States Index (FSI) 2020 Fragility in the World.

[10] Bush A, Keylock J. Nutrition Works. Strengthening Integration of Nutrition within Health Sector Programmes An Evidence-based Planning Resource. European Commission. https://www.nutritionworks.org.uk/technical-guidance-and-research/strengthening-integration-of-nutrition-within-health-sector-programmes-an-evidence-based-planning-resource/.

[11] Sterne JAC, Savović J, Page MJ, Elbers RG, Blencowe NS, Boutron I, Cates CJ, Cheng H-Y, Corbett MS, Eldridge SM, Hernán MA, Hopewell S, Hróbjartsson A, Junqueira DR, Jüni P, Kirkham JJ, Lasserson T, Li T, McAleenan A, Reeves BC, Shepperd S, Shrier I, Stewart LA, Tilling K, White IR, Whiting PF, Higgins JPT (2019). RoB 2: a revised tool for assessing risk of bias in randomised trials. BMJ.

[12] Sterne J, Hernán M, McAleenan A, Reeves B, Higgins J. Chapter 25: Assessing risk of bias in a non-randomized study | Cochrane Training. Cochrane Handb Syst Rev Interv. 2019; https://training.cochrane.org/handbook/current/chapter-25.

[13] Critical Appraisal Skills Programme (2019). CASP Qualitative & Quantitative Studies Checklist.

[14] Arifeen SE, Hoque DE, Akter T, Rahman M, Hoque ME, Begum K, Ahmed S (2009). Effect of the integrated management of childhood illness strategy on childhood mortality and nutrition in a rural area in Bangladesh: a cluster randomised trial. Lancet.

[15] Armstrong SJ, Bryce J, de Savigny D, Lambrechts T, Mbuya C, Mgalula L, Wilczynska K (2004). The effect of integrated management of childhood illness on observed quality of care of under-fives in rural Tanzania. Health Policy Plan.

[16] Bhandari N, Mazumder S, Taneja S, Sommerfelt H. neonatal and childhood illness (IMNCI) programme on neonatal and infant mortality: cluster randomised controlled trial. BMJ. 344(mar21 1):e1634. 10.1136/bmj.e1634. 10.1136/bmj.e1634 PMC3309879 22438367

[17] Bryce J, Gouws E, Adam T, Black RE, Schellenberg JA, Manzi F (2005). Improving quality and efficiency of facility-based child health care through integrated management of childhood illness in Tanzania. Health Policy Plan.

[18] El Arifeen S, Blum LS, Hoque DE, Chowdhury EK, Khan R, Black RE, Bryce J (2004). Integrated management of childhood illness (IMCI) in Bangladesh: Early findings from a cluster-randomised study. Lancet.

[19] Friedman L, WoLFheim C (2014). Linking nutrition & (integrated) community case management. A review of operational experiences.

[20] Masanja H, Schellenberg JA, De Savigny D, Mshinda H, Victora CG (2005). Impact of integrated Management of Childhood Illness on inequalities in child health in rural Tanzania. Health Policy Plan.

[21] Mazumder S, Taneja S, Bahl R, Mohan P, Strand TA, Sommerfelt H (2014). Effect of implementation of integrated management of neonatal and childhood illness programme on treatment seeking practices for morbidities in infants: cluster randomised trial. BMJ.

[22] Miller NP, Amouzou A, Tafesse M, Hazel E, Legesse H, Degefie T, Bryce J (2014). Integrated community case management of childhood illness in Ethiopia: implementation strength and quality of care. Am J Trop Med Hyg.

[23] Rasanathan K, Muñiz M, Bakshi S, Kumar M, Solano A, Kariuki W, George A, Sylla M, Nefdt R, Young M, Diaz T. Community case management of childhood illness in sub-Saharan Africa - findings from a cross-sectional survey on policy and implementation. J Glob Health. 2014;4(2):020401. 10.7189/jogh.04.020401. 10.7189/jogh.04.020401 PMC4267096 25520791

[24] Schellenberg JRA, Adam T, Mshinda H, Masanja H, Kabadi G, Mukasa O, Wilczynska K (2004). Effectiveness and cost of facility-based integrated management of childhood illness (IMCI) in Tanzania. Lancet.

[25] Taneja S, Bahl S, Mazumder S, Martines J, Bhandari N, Bhan MK. Impact on inequities in health indicators: effect of implementing the integrated management of neonatal and childhood illness programme in Haryana, India. J Glob Health. 2015;5(1). 10.7189/jogh.05.010401. 10.7189/jogh.05.010401 PMC4306296 25674350

[26] Aguayo VM, Agarwal V, Agnani M, Agrawal DD, Bhambhal S, Rawat AK, Singh K (2013). Integrated program achieves good survival but moderate recovery rates among children with severe acute malnutrition in India. Am J Clin Nutr.

[27] Amadi B, Imikendu M, Sakala M, Banda R, Kelly P (2016). Integration of HIV care into community management of acute childhood malnutrition permits good outcomes: retrospective analysis of three years of a programme in Lusaka. PLoS One.

[28] Brits H, Joubert G, Eyman K, De Vink R, Lesaoana K, Makhetha S, Moeketsi K (2017). An assessment of the integrated nutrition programme for malnourished children aged six months to five years at primary healthcare facilities in Mangaung, Free State, South Africa. S Afr Fam Pract.

[29] Deconinck H, Hallarou ME, Pesonen A, Gérard JC, Criel B, Donnen P, Macq J (2016). Understanding factors that influence the integration of acute malnutrition interventions into the national health system in Niger. Health Policy and Plan.

[30] Kouam CE, Delisle H, Ebbing HJ, Israël AD, Salpéteur C, Aïssa MA, Ridde V (2014). Perspectives for integration into the local health system of community-based management of acute malnutrition in children under 5 years: a qualitative study in Bangladesh. Nutr J.

[31] Puett C, Alderman H, Sadler K, Coates J (2015). ‘Sometimes they fail to keep their faith in us’: community health worker perceptions of structural barriers to quality of care and community utilisation of services in Bangladesh. Maternal Child Nutr.

[32] Puett C, Coates J, Alderman H, Sadler K (2013). Quality of care for severe acute malnutrition delivered by community health workers in southern Bangladesh. Maternal Child Nutr.

[33] Sadler K, Puett C, Mothabbir G, Myatt M (2011). Community case management of severe acute malnutrition in southern Bangladesh.

[34] Tadesse E, Worku A, Berhane Y, Ekström EC. An integrated community-based outpatient therapeutic feeding programme for severe acute malnutritionin rural Southern Ethiopia: Recovery, fatality, and nutritional status after discharge. Matern Child Nutr. 2018;14(2):e12519. 10.1111/mcn.12519. Epub 2017 Oct 10. 10.1111/mcn.12519 PMC5900575 29024381

[35] Doherty T, Chopra M, Tomlinson M, Oliphant N, Nsibande D, Mason J (2010). Moving from vertical to integrated child health programmes: experiences from a multi-country assessment of the child health days approach in Africa. Tropical Med Int Health.

[36] Palmer AC, Diaz T, Noordam AC, Dalmiya N (2013). Evolution of the child health day strategy for the integrated delivery of child health and nutrition services. Food Nutr Bull.

[37] Anand A, Luman ET, O'Connor PM (2012). Building on success— potential to improve coverage of multiple health interventions through integrated delivery with routine childhood vaccination. J Infect Dis.

[38] Baqui A, Williams EK, Rosecrans AM, Agrawal PK, Ahmed S, Darmstadt GL, Ahuja RC (2008). Impact of an integrated nutrition and health programme on neonatal mortality in rural northern India. Bull World Health Organ.

[39] Ching P, Birmingham M, Goodman T, Sutter R, Loevinsohn B (2000). Childhood mortality impact and costs of integrating vitamin a supplementation into immunization campaigns. Am J Public Health.

[40] Hodges MH, Sesay FF, Kamara HI, Nyorkor ED, Bah M, Koroma AS, Katcher HI (2015). Integrating vitamin a supplementation at 6 months into the expanded program of immunization in Sierra Leone. Matern Child Health J.

[41] Klemm RD, Villate EE, Tuazon-Lopez C, Ramos AC (1996). Coverage and impact of adding vitamin a capsule (VAC) distribution to annual national immunisation day in the Philippines.

[42] Ropero-Álvarez AM, Kurtis HJ, Danovaro-Holliday MC, Ruiz-Matus C, Tambini G (2012). Vaccination week in the Americas: an opportunity to integrate other health services with immunization. J Infect Diseas.

[43] Fernandez-Rao S, Hurley KM, Nair KM, Balakrishna N, Radhakrishna KV, Ravinder P, Tilton N, Harding KB, Reinhart GA, Black MM (2014). Integrating nutrition and early child-development interventions among infants and preschoolers in rural India. Ann N Y Acad Sci.

[44] Gowani S, Yousafzai AK, Armstrong R, Bhutta ZA (2014). Cost effectiveness of responsive stimulation and nutrition interventions on early child development outcomes in Pakistan. Ann N Y Acad Sci.

[45] Yousafzai AK, Rasheed MA, Rizvi A, Armstrong R, Bhutta ZA (2014). Effect of integrated responsive stimulation and nutrition interventions in the lady health worker programme in Pakistan on child development, growth, and health outcomes: a cluster-randomised factorial effectiveness trial. Lancet.

[46] Grellety E, Babakazo P, Bangana A, Mwamba G, Lezama I, Zagre NM, Ategbo E-A (2017). Effects of unconditional cash transfers on the outcome of treatment for severe acute malnutrition (SAM): a cluster-randomised trial in the Democratic Republic of the Congo. BMC Med.

[47] Berti PR, Mildon A, Siekmans K, Main B, MacDonald C (2010). An adequacy evaluation of a 10-year, four-country nutrition and health programme. Int J Epidemiol.

[48] Fagerli K, O'Connor K, Kim S, Kelley M, Odhiambo A, Faith S, Otieno R, Nygren B, Kamb M, Quick R (2017). Impact of the integration of water treatment, hygiene, nutrition, and clean delivery interventions on maternal health service use. Am J Trop Med Hyg.

[49] Grossmann VM, Turner BS, Snyder D, Stewart RD, Bowen T, Cifuentes AA, Cliff C (2015). Zinc and vitamin supplementation in an under-5 indigenous population of Guatemala: influence of lay health promoters in decreasing incidence of diarrhea. J Transcult Nurs.

[50] Guyon AB, Quinn VJ, Hainsworth M, Ravonimanantsoa P, Ravelojoana V, Rambeloson Z, Martin L (2009). Implementing an integrated nutrition package at large scale in Madagascar: the essential nutrition actions framework. Food Nutr Bull.

[51] Nguyen PH, Kim SS, Sanghvi T, Mahmud Z, Tran LM, Shabnam S (2017). Integrating nutrition interventions into an existing maternal, neonatal, and child health program increased maternal dietary diversity, micronutrient intake, and exclusive breastfeeding practices in Bangladesh: results of a cluster-randomized program evaluation. J Nutr.

[52] Parikh K, Marein-Efron G, Huang S, O'Hare G, Finalle R, Shah SS (2010). Nutritional status of children after a food-supplementation program integrated with routine health care through mobile clinics in migrant communities in the Dominican Republic. Am J Trop Med Hyg.

[53] Saiyed F, Seshadri S (2000). Impact of the integrated package of nutrition and health services. Indian J Pediatr.

[54] Singh V, Ahmed S, Dreyfuss ML, Kiran U, Chaudhery DN, Srivastava VK, Santosham M (2017). Non-governmental organization facilitation of a community-based nutrition and health program: effect on program exposure and associated infant feeding practices in rural India. PLoS One.

[55] Sivanesan S, Kumar A, Kulkarni MM, Kamath A, Shetty A (2016). Utilization of integrated child development services (ICDS) scheme by child beneficiaries in coastal Karnataka India. Indian J Community Health.

[56] Tandon B (1989). Nutritional interventions through primary health care: impact of the ICDS projects in India. Bull World Health Organ.

[57] Head J, Pachón H, Tadesse W, Tesfamariam M, Freeman MC (2019). Integration of water, sanitation, hygiene and nutrition programming is associated with lower prevalence of child stunting and fever in Oromia, Ethiopia. Afr J Food Agri Nutr Dev.

