# Supplementary material for: Best practices and opportunities for integrating nutrition specific into nutrition sensitive interventions in fragile contexts: a systematic review
Source: BMC Nutr. 2021 Jul 29;7:46. doi: 10.1186/s40795-021-00443-1 (PMC8320180; doi:10.1186/s40795-021-00443-1)
Supplement: Supplementary file 1 — Additional file 1: Table S1. PRISMA guideline. Table S2. Pubmed search. Table S3. Risk of bias of the included studies. [file 40795_2021_443_MOESM1_ESM.docx]

**Supplementary materials**

**Best Practices and Opportunities for Integrating Nutrition Specific into Nutrition Sensitive Interventions** **in Fragile Contexts: A Systematic Review**

Leila H Abdullahi^1*^, Gilbert K Rithaa, Bonface Muthomi^3^, Florence Kyallo^4^, Clementina Ngina^5^, Mohamed A Hassan^6^, Mohamed A Farah^6^

^1^ African Institute for Development Policy (AFIDEP), Kenya

^2^ Scaling Up Nutrition (SUN-FP), Somalia

^3^ Gretsa University, Kenya

^4^ Jomo Kenyatta University of Agriculture and Technology, Kenya

^5^ Freelance, Nutrition consultant, Kenya & Somalia

^6^ Office of the Prime Minister, Somalia

**Corresponding author;**

*Leila Hussein Abdullahi

Leylaz@live.co.za

**Supplementary table 1:** **PRISMA guideline**

| **Section/topic** | **#** | **Checklist item** | **Reported on page #** |
| --- | --- | --- | --- |
| **TITLE** | | |  |
| Title | 1 | Identify the report as a systematic review, meta-analysis, or both. | 1 |
| **ABSTRACT** | | |  |
| Structured summary | 2 | Provide a structured summary including, as applicable: background; objectives; data sources; study eligibility criteria, participants, and interventions; study appraisal and synthesis methods; results; limitations; conclusions and implications of key findings; systematic review registration number. | 2 |
| **INTRODUCTION** | | |  |
| Rationale | 3 | Describe the rationale for the review in the context of what is already known. | 5/6 |
| Objectives | 4 | Provide an explicit statement of questions being addressed with reference to participants, interventions, comparisons, outcomes, and study design (PICOS). | 6 |
| **METHODS** | | |  |
| Protocol and registration | 5 | Indicate if a review protocol exists, if and where it can be accessed (e.g., Web address), and, if available, provide registration information including registration number. | CRD42020209730 |
| Eligibility criteria | 6 | Specify study characteristics (e.g., PICOS, length of follow-up) and report characteristics (e.g., years considered, language, publication status) used as criteria for eligibility, giving rationale. | 7/10 |
| Information sources | 7 | Describe all information sources (e.g., databases with dates of coverage, contact with study authors to identify additional studies) in the search and date last searched. | 11 |
| Search | 8 | Present full electronic search strategy for at least one database, including any limits used, such that it could be repeated. | Suppl 2 |
| Study selection | 9 | State the process for selecting studies (i.e., screening, eligibility, included in systematic review, and, if applicable, included in the meta-analysis). | 11 |
| Data collection process | 10 | Describe method of data extraction from reports (e.g., piloted forms, independently, in duplicate) and any processes for obtaining and confirming data from investigators. | 11 |
| Data items | 11 | List and define all variables for which data were sought (e.g., PICOS, funding sources) and any assumptions and simplifications made. | 11 |
| Risk of bias in individual studies | 12 | Describe methods used for assessing risk of bias of individual studies (including specification of whether this was done at the study or outcome level), and how this information is to be used in any data synthesis. | 12 |
| Summary measures | 13 | State the principal summary measures (e.g., risk ratio, difference in means). | 12 |
| Synthesis of results | 14 | Describe the methods of handling data and combining results of studies, if done, including measures of consistency (e.g., I^2^) for each meta-analysis. | 12 |
| Risk of bias across studies | 15 | Specify any assessment of risk of bias that may affect the cumulative evidence (e.g., publication bias, selective reporting within studies). | 12 |
| Additional analyses | 16 | Describe methods of additional analyses (e.g., sensitivity or subgroup analyses, meta-regression), if done, indicating which were pre-specified. | 12 |
| **RESULTS** | | |  |
| Study selection | 17 | Give numbers of studies screened, assessed for eligibility, and included in the review, with reasons for exclusions at each stage, ideally with a flow diagram. | 13 |
| Study characteristics | 18 | For each study, present characteristics for which data were extracted (e.g., study size, PICOS, follow-up period) and provide the citations. | Table 1; 15/18 |
| Risk of bias within studies | 19 | Present data on risk of bias of each study and, if available, any outcome level assessment (see item 12). | 18 & appendix2 |
| Results of individual studies | 20 | For all outcomes considered (benefits or harms), present, for each study: (a) simple summary data for each intervention group (b) effect estimates and confidence intervals, ideally with a forest plot. | 19 |
| Synthesis of results | 21 | Present results of each meta-analysis done, including confidence intervals and measures of consistency. | 19/22 |
| Risk of bias across studies | 22 | Present results of any assessment of risk of bias across studies (see Item 15). | 19 |
| Additional analysis | 23 | Give results of additional analyses, if done (e.g., sensitivity or subgroup analyses, meta-regression [see Item 16]). | 23/23 |
| **DISCUSSION** | | |  |
| Summary of evidence | 24 | Summarize the main findings including the strength of evidence for each main outcome; consider their relevance to key groups (e.g., healthcare providers, users, and policy makers). | 28/29 |
| Limitations | 25 | Discuss limitations at study and outcome level (e.g., risk of bias), and at review-level (e.g., incomplete retrieval of identified research, reporting bias). | 28/29 |
| Conclusions | 26 | Provide a general interpretation of the results in the context of other evidence, and implications for future research. | 29 |
| **FUNDING** | | |  |
| Funding | 27 | Describe sources of funding for the systematic review and other support (e.g., supply of data); role of funders for the systematic review. | 30 |

*From:*  Moher D, Liberati A, Tetzlaff J, Altman DG, The PRISMA Group (2009). Preferred Reporting Items for Systematic Reviews and Meta-Analyses: The PRISMA Statement. PLoS Med 6(7): e1000097. doi:10.1371/journal.pmed1000097

**Supplementary table 2: Pubmed search**

| **Table 1: Search Strategy** | |
| --- | --- |
| **Subject** | **Search Terms** |
| Integration | integrat* care OR "integration of care" OR integrat* services OR "integration of services" OR integrat* programmes OR integrat* programs OR “integration of programmes” OR “integration of programs” OR integrat* service delivery OR “integration of service delivery” OR integrat* services OR “integration of services” OR integrat* delivery OR integrat* management OR “integration of  management”  OR  coordinat* care OR "coordination of care" OR coordinat* services OR "coordination of services" OR coordinat* programmes OR coordinat* programs OR “coordination of programmes” OR “coordination of programs” OR coordinat* service delivery OR “coordination of service delivery” OR co ordinat* services OR “coordination of services” OR coordinat* delivery OR coordinat*  management OR “coordination of management” OR co-ordinat* care OR "co-ordination of care" OR co-ordinat* services OR "co-ordination of  services" OR co-ordinat* programmes OR co-ordinat* programs OR “co-ordination of programmes” OR “co-ordination of programs” OR co-ordinat* service delivery OR “co-ordination of service delivery” OR co-ordinat* services OR “co-ordination of services” OR co-ordinat* delivery OR coordinat* management OR “co-ordination of management”  OR  horizontal care OR vertical care OR horizontal services OR vertical services OR horizontal programmes OR horizontal programs OR vertical programmes OR vertical programs OR horizontal service delivery OR vertical service delivery OR horizontal services OR vertical services OR horizontal delivery OR vertical management OR vertical management |
| Interventions | 'nutrition intervention' OR 'nutrition specific program' OR 'nutrition sensitive program' OR 'nutrition specific intervention' OR 'nutrition sensitive intervention' |
| Nutrition outcomes | "nutritional status" OR "nutritional outcomes" OR malnutrition OR "diet* diversity" OR micronutrient* OR growth OR anthropometr*, |

**Supplementary table 3: Risk of bias of the included studies**

**Quantitative observational studies**

| Study | Was the study's target population a close representation of the national population ? | Was the sampling frame a true or close representation of the target population? | Was some form of random selection used to select the sample? | Was the likelihood of nonresponse bias minimal? | Were data collected directly from the subjects (as opposed to a proxy)? | Was an acceptable case definition used in the study? | Was the study instrument that measured the parameter of interest shown to have validity and reliability? | Was the same mode of data collection used for all subjects? | Was the length of the shortest period for the parameter of interest appropriate? | Were the numerator(s) and denominator(s) for the parameter of interest appropriate? | Reviewers comment |
| --- | --- | --- | --- | --- | --- | --- | --- | --- | --- | --- | --- |
| Armstrong et al 2004 [15] | Not clear | No | No | Not clear | No | Yes | Yes | Yes | Yes | Yes | Moderate |
| Friedman & Wolfheim 2014 [19] | Yes | Yes | No | Not clear | No | Yes | Yes | Yes | Yes | Yes | Low risk |
| Masanja et al 2005 [20] | Yes | Yes | Not clear | Not clear | No | Yes | Yes | Yes | Yes | Yes | Moderate |
| Miller et al 2014 [22] | Yes | Yes | Yes | Not clear | Yes | Yes | Yes | Yes | Not clear | Yes | Moderate |
| Rasanathan et al 2014 [23] | Yes | Yes | No | Not clear | Yes | Yes | Yes | Yes | Yes | Yes | Low risk |
| Schellenberg et al 2004 [24] | Yes | Yes | Yes | Not clear | Yes | Yes | Yes | Yes | Not clear | Yes | Moderate risk |
| Aguayo et al 2013 [26] | Yes | Yes | Yes | Not clear | Yes | Yes | Yes | Yes | Not clear | Yes | Moderate risk |
| Amadi et al 2016 [27] | Not clear | No | No | Not clear | No | Yes | Yes | Yes | Not clear | Yes | High risk |
| Brits et al 2017 [28] | Yes | Yes | No | Not clear | No | Yes | Yes | Yes | Yes | Yes | Low risk |
| Puett et al 2013 [32] | Yes | Yes | Yes | Not clear | Yes | Yes | Yes | Yes | Yes | Yes | Low risk |
| Sadler et al 2011 [33] | Yes | Yes | Yes | Not clear | Yes | Yes | Yes | Yes | Yes | Yes | Low risk |
| Tadesse et al 2017 [34] | Yes | Yes | Yes | Not clear | Yes | Yes | Yes | Yes | Not clear | Yes | Moderate risk |
| Doherty et al 2010 [35] | Not clear | No | No | Not clear | No | Yes | Yes | Yes | Not clear | Yes | High risk |
| Palmer et al 2013 [36] | Yes | Yes | Yes | Not clear | Yes | Yes | Yes | Yes | Not clear | Yes | Moderate risk |
| Anand et al 2012 [37] | Yes | Yes | Not clear | Not clear | No | Yes | Yes | Yes | Yes | Yes | Moderate risk |
| Ching et al 2000 [39] | Yes | Yes | Yes | Not clear | Yes | Yes | Yes | Yes | Yes | Yes | Low risk |
| Klemm et al 1996 [41] | Yes | Yes | Not clear | Not clear | No | Yes | Yes | Yes | Yes | Yes | Moderate risk |
| Ropero-Álvarez et al 2012 [42] | Yes | Yes | No | Not clear | No | Yes | Yes | Yes | Not clear | Yes | Moderate risk |
| Berti et al 2010 [47] | Not clear | No | No | Not clear | No | Yes | Yes | Yes | Not clear | Yes | High risk |
| Fagerli et al 2017 [48] | Yes | Yes | Yes | Not clear | Yes | Yes | Yes | Yes | Not clear | Yes | Moderate risk |
| Parikh et al 2010 [52] | Yes | Yes | Not clear | Not clear | No | Yes | Yes | Yes | Yes | Yes | Low risk |
| Saiyed & Seshadri 2000 [53] | Yes | Yes | Yes | Not clear | Yes | Yes | Yes | Yes | Not clear | Yes | Moderate risk |
| Sivanesan et al 2016 [55] | Yes | Yes | Not clear | Not clear | No | Yes | Yes | Yes | Yes | Yes | Moderate risk |
| Tandon, 1989 [56] | Yes | Yes | Yes | Not clear | Yes | Yes | Yes | Yes | Yes | Yes | Low risk |
| Head Jeniffer 1999 [57] | Yes | Yes | Not clear | Not clear | No | Yes | Yes | Yes | Yes | Yes | Moderate risk |

**Individual and cluster randomized intervention studies**

| Study | Selection bias (allocation concealment, allocation sequence ) | Performance bias | Detection bias | Attrition bias? | Reporting bias? | Are study results valid? | Others (specify) | Reviewers comment |
| --- | --- | --- | --- | --- | --- | --- | --- | --- |
| Arifeen et al 2009 [14] | Low risk | Unclear | Low risk | Low risk | Low risk | Low risk | Unclear | Moderate risk |
| Bhandari et al 2012 [16] | Low risk | Low risk | Unclear | Low risk | Low risk | Low risk | Unclear | Moderate risk |
| El Arifeen et al 2004 [18] | High risk | Unclear | Low risk | Low risk | Low risk | Low risk | Unclear | Moderate risk |
| Mazumder et al 2014 [21] | Low risk | Unclear | Low risk | Low risk | Low risk | Low risk | Unclear | Moderate risk |
| Taneja et al 2015 [25] | High risk | Unclear | Low risk | Low risk | Low risk | Low risk | Unclear | Moderate risk |
| Fernandez‐Rao et al 2014 [43] | Low risk | Low risk | Unclear | Low risk | Low risk | Low risk | Unclear | Moderate risk |
| Gowani et al 2014 [44] | High risk | Unclear | Low risk | Low risk | Low risk | Low risk | Unclear | Moderate risk |
| Yousafzai et al 2014 [45] | Low risk | Unclear | Low risk | Low risk | Low risk | Low risk | Unclear | Moderate risk |
| Grellety et al 2017 [46] | High risk | Unclear | Low risk | Low risk | Low risk | Low risk | Unclear | Moderate risk |
| Nguyen et al 2017 [51] | Low risk | Low risk | Unclear | Low risk | Low risk | Low risk | Unclear | Moderate risk |

**Non-randomized intervention studies**

| Study | **Pre-intervention** | | **At intervention** | **Post-intervention** | | | | Reviewers comment |
| --- | --- | --- | --- | --- | --- | --- | --- | --- |
|  | Bias due to confounding | Bias in selection of participants into the study | Bias in classification of interventions | Bias due to deviations from intended interventions | Bias due to missing data | Bias in measurement of outcomes | Bias in selection of the reported result |  |
| Bryce et al 2005 [17] | Low risk | Unclear | Low risk | Low risk | Low risk | Low risk | Unclear | Moderate risk |
| Baqui et al 2008 [38] | Low risk | Low risk | Unclear | Low risk | Low risk | Low risk | Unclear | Moderate risk |
| Hodges et al 2015 [40] | High risk | Unclear | Low risk | Low risk | Low risk | Low risk | Unclear | Moderate risk |
| Grossmann et al 2015 [49] | Low risk | Low risk | Unclear | Low risk | Low risk | Low risk | Unclear | Moderate risk |
| Guyon et al 2009 [50] | Low risk | Low risk | Unclear | Low risk | Low risk | Low risk | Unclear | Moderate risk |
| Singh et al 2017 [54] | High risk | Unclear | Low risk | Low risk | Low risk | Low risk | Unclear | Moderate risk |

**Qualitative studies**

| Study | Is a qualitative approach appropriate? | Is the study clear in what it seeks to do? | How defensible/rigorous is the research design/methodology? | How clear and coherent is the reporting of ethics? | How well was the data collection carried out? | Is the role of the researcher clearly described? | Is the context clearly described? | Were the methods reliable? | Is the data analysis sufficiently rigorous? | Is the data 'rich'? | Is the analysis reliable? | Are the findings convincing? | Are the findings relevant to the aims of the study? | Reviewers comment |
| --- | --- | --- | --- | --- | --- | --- | --- | --- | --- | --- | --- | --- | --- | --- |
| Deconinck et al 2016 [29] | Yes | Yes | Defensible | Appropriate | Appropriately | Clearly described | Clearly described | Reliable | Not sure | Rich | Reliable | Convincing | Relevant | Low risk |
| Kouam et al 2014 [30 | Yes | Yes | Defensible | Appropriate | Appropriately | Clearly described | Clearly described | Reliable | Not sure | Rich | Reliable | Convincing | Relevant | Low risk |
| Puett et al 2015 [31] | Yes | Yes | Defensible | Appropriate | Appropriately | Clearly described | Clearly described | Reliable | Not sure | Rich | Reliable | Convincing | Relevant | Low risk |
